# Clinical Characteristics and Potential Risk Factors Associated with the SARS-CoV-2 Infection: Survey on a Health Care Workers (HCWs) Population in Northern Italy

**DOI:** 10.3390/ijerph19138194

**Published:** 2022-07-04

**Authors:** Viola Novelli, Federico Fassio, Guido Resani, Martino Bussa, Alessandro Durbano, Alessandro Meloni, Giovanni Oliva, Sara Cutti, Daniela Girardi, Anna Odone, Simona Villani, Carlo Marena, Alba Muzzi, Maria Cristina Monti

**Affiliations:** 1 Medical Direction, Fondazione IRCCS Policlinico San Matteo, 27100 Pavia, Italy; v.novelli@smatteo.pv.it (V.N.); guido.resani01@universitadipavia.it (G.R.); ale.durbano@gmail.com (A.D.); s.cutti@smatteo.pv.it (S.C.); daniela.girardi01@universitadipavia.it (D.G.); c.marena@smatteo.pv.it (C.M.); a.muzzi@smatteo.pv.it (A.M.); 2 Unit of Biostatistics and Clinical Epidemiology, Department of Public Health, Experimental and Forensic Medicine, University of Pavia, 27100 Pavia, Italy; bussamartino@gmail.com (M.B.); simona.villani@unipv.it (S.V.); cristina.monti@unipv.it (M.C.M.); 3 Unit of Hygiene, Department of Public Health, Experimental and Forensic Medicine, University of Pavia, 27100 Pavia, Italy; alessandro.meloni01@universitadipavia.it (A.M.); giovanni.oliva01@universitadipavia.it (G.O.); anna.odone@unipv.it (A.O.)

**Keywords:** COVID-19, healthcare workers, survey, SARS-CoV-2

## Abstract

During the two years of the COVID-19 pandemic, more than 400 million cases all over the world have been identified. Health care workers were among the first to deal with this virus and consequently a high incidence of infection was reported in this population. The aim of the survey was to investigate health care workers’ (HCWs) clinical characteristics and potential risk factors associated with the SARS-CoV-2 infection in a referral hospital in Northern Italy after the first and second waves of the pandemic. We administered a questionnaire during the flu vaccination campaign that took place at the end of 2020; among 1386 vaccinated HCWs, data was collected and analyzed for 1065 subjects. 182 HCWs (17%) declared that they had tested positive on at least a molecular or a serological test since the beginning of the pandemic. Comparing the infected vs. not infected HCWs, median age, BMI, smoking habit, presence of hypertension or other comorbidities were not significantly different, while having worked in a COVID ward was associated with the infection (OR_adj_ = 1.54, 95% CI: 1.07–2.20). Respondents declared that more than 70% of contacts occurred in the hospital with patients or colleagues, while about 15% in domestic environments. Among the infected, the most reported symptoms were fever (62.1%), asthenia (60.3%), anosmia/ageusia (53.5%), arthralgia/myalgia (48.3%), headache or other neurological symptoms (46.6%), cough (43.1%) and flu-like syndrome (41.4%). The percentage of subjects who have been infected with SARS-CoV-2 seems to be higher in HCWs than in the general population; hence, in hospitals, protective measures and preventive strategies to avoid the spreading of the contagion remain crucial.

## 1. Introduction

The new coronavirus SARS-CoV-2, identified in China in late December 2019, is the cause of COVID-19 respiratory syndrome and has quickly spread around the world. As of 31 December 2020, confirmed cases worldwide were just under 83 million and confirmed deaths were approximatively 2 million, while in Italy the death toll exceeded 73,000. One year later, the total numbers had almost tripled: 280 million of cases and 5.4 million deaths [1].

The main clinical manifestations generally occur between four to five days after exposure, and the incubation period can last up to 14 days. Common symptoms are nonspecific and include fever, cough, fatigue, and shortness of breath [2]. There is also a multiorgan involvement, with anosmia and ageusia or other alterations of the central and peripheral nervous system, cardiac issues (myocardial damage, arrhythmia), gastrointestinal involvement(nausea, vomiting, diarrhea), and cutaneous (petechiae, vesicles) and hematological (lymphopenia and thrombocytopenia) complications [3].

Since the beginning of the pandemic, health care workers (HCWs) had to face an emergency situation with little knowledge about this new infection. Therefore, in the first months of 2020, the percentage of contagion was very high compared to the general population (even reaching 12%), and it gradually reduced until it settled around 3–4% [4]. A rigorous follow-up of HCWs was then required in order to improve contagion prevention and to develop a model that was also valid for public health. Many hospitals investigated possible risk factors and clinical manifestations in workers who contracted the disease [5], through the use of company health databases or via surveys [6,7].

Many international efforts have been made to safeguard the health of hospital workers, encouraging the use of PPE and updating professionals on the most recent discoveries about the new coronavirus [8]; however, the number of infections and deaths among health personnel remained significant [9,10]. At the beginning of the pandemic, there might have been a potential underestimation of the percentage infected due to poor resources and insufficient knowledge to correctly identify deaths linked to COVID-19 [11].

In Lombardy, the university hospital IRCCS Policlinico San Matteo was one of the major locations for the treatment of the SARS-CoV-2infection. The first confirmed Italian case of COVID-19 was hospitalized on 21 February 2020 in its Intensive Care Unit. The cumulative incidence of the SARS-CoV-2 infection among San Matteo hospital workers, in May 2020, was 3.54% [12]. Following preliminary analysis carried out by occupational doctors for the internal surveillance, we decided to collect additional data by administering a questionnaire.

The principal aim of this study was to describe how many HCWs reported having contracted the SARS-CoV-2 infection and the exposure factors that may have played a role in susceptibility to infection in a potentially vulnerable population (i.e., healthcare professionals) as well as clinical characteristics associated with the infection itself.

## 2. Materials and Methods

A single cross-sectional study was carried out on HCWs at the IRCCS Policlinico San Matteo in Pavia, Lombardy. When the survey was carried out, the hospital workers numbered just over 4600. Data were collected through a questionnaire that consisted of 29 questions regarding socio-demographic characteristics, job qualification and work department, alcohol intake and smoking habits, the correct use of personal protective equipment (PPE), general health status, previous positivity to SARS-CoV-2 infection and related symptoms, and infection risk perception. It was created on the basis of a COVID-19 symptom triage questionnaire that had been administered to symptomatic workers or close contacts.

The SARS-CoV-2 positivity among the hospital workers undergoing swabs, in May 2020, was 11.3%; since an infection increase was expected (according to world data), the minimum sample size was calculated by estimating a cumulative incidence of 20% ± 5% and a confidence level of 95%; assuming, moreover, a response rate of 50% and setting hospital total workers as population size (N = 4600), at least 467 subjects would have been needed.

Hospital employees were invited to participate in the survey when they showed up to join the flu vaccination campaign between 27 November 2020 and 20 December 2020. Participation was voluntary. Questionnaires were returned in a sealed urn. Information was entered in an anonymized database by doctors of the hospital with the support of the Biostatistics and Epidemiology Unit of the University of Pavia.

### 2.1. Outcome and Variables

Subjects who declared at least one positive serological/molecular test were considered to have a history of infection [13], while those without any positivity were considered infection-naive (COVID+/COVID−). A scale, ranging from 0 to 5, was used to assess the degree of risk perception of the SARS-CoV-2 infection. Body mass index (BMI) was calculated on self-reported weight and height (kg/m^2^).

Data relating to symptoms following the SARS-CoV-2 infection were collected only for subjects who tested positive on a molecular test, as serological positivity could be associated with an unnoticed previous infection.

### 2.2. Statistical Analysis

The characteristics of subjects who completed the questionnaire were described using mean and standard deviation (or median and interquartile range (IQR) if the assumption of normality was not respected) for quantitative variables and frequency for qualitative variables. Subjects who had declared a SARS-CoV-2 infection were compared to workers who had not contracted it. A chi-square test (χ^2^) was applied for qualitative variables and a *t*-test or Mann-Whitney test was used for quantitative variables. Those variables showing a significance <0.1 in the exploratory analysis were included in the multiple logistic regression model (SARS-CoV-2 infection COVID+/COVID− was the dependent variable). Candidate confounders, such as smoking status and BMI, were not included in the model because they were not primarily associated with the outcome. Sex and age (in years) were forced in the model because of their clinical interest. For working roles, administrator and researcher were considered as a reference category. We decided to exclude the risk perception of the infection from the model, albeit significant, in order to exploratory analysis, as its evaluation could have been influenced by previous contagion and therefore could not be a real predictive factor. A *p*-value < 0.05 was considered significant in secondary analysis. Crude and adjusted odds ratios (OR_adj_) and 95% confidence intervals (95% CI) were used as the measure of effect and precision, respectively. McFadden pseudo-R^2^ was reported as the measure of explained variance and the Hosmer-Lemeshow test was used for the goodness-of-fit. Data were processed using STATA software (2019, release 16.1: StataCorp, College Station, TX, USA).

## 3. Results

Subjects who joined the vaccination campaign totaled 1386; among them, 1093 took part in the survey (response rate: 79%). Of these, data relating to 1065 health workers were analyzed after the exclusion of poorly filled out questionnaires (i.e., the absence of more than 50% of the required information). Two-thirds of subjects were female and the median age was 42 years (see Table 1). 98.1% of the responders was Caucasian. Most of the sample was composed of physicians (47.3%; N = 504), while 25.2% were made up of nurses or midwives (N = 268), 15.8% were administrative personnel/researchers or fellows (N = 168), 6.0% were technical operators (N = 64) and 5.7% were OSS (social health workers) (N = 61).

Overall, 17.1% (N = 182) were declared to have been positive for COVID-19: 55 reported only molecular test positivity, 66 only tested positive serologically, 61 both tests positivity (10.9% of the sample was positive using a molecular test and 12.7% using a serological one). There did not appear to be a significant difference in infection by working role (*p* = 0.085).

The BMI was found to be on average 23.6 ± 4.3 kg/m^2^, with no difference between those who had contracted the infection and those who did not (Table 1). Just over twenty percent of subjects reported being active smokers, with no differences between subjects with previous infections and the uninfected ones. 36.5% of SARS-CoV-2 positive subjects drank alcohol, compared to 43.9% of those who were not infected. Over ninety-six percent of subjects declared to have received correct and adequate information about the use of PPE, without differences between the infected and those that were not (Table 1). The percentage of subjects who had worked in contact with COVID positive patients was significantly higher (*p* = 0.009) among those who contracted the infection than among who did not contract it. As reported in Table 1, the proportion was reversed. The use of public transport and parenting of school-age children did not have a significant association with contracting the coronavirus (Table 1).

The multiple logistic regression model includes sex, age, alcohol intake, having worked in contact with SARS-CoV-2 positive patients, and job position as independent variables (Table 2). Having worked in a ward with COVID positive subjects significantly increased the odds of COVID infection, independently from the other variables included in the model (OR_adj_ = 1.54; *p* = 0.020). Being a nurse/midwife or OSS increased the odds of COVID infection compared to an administrative/researcher role in the univariate model, while after adjusting by sex, age and alcohol intake and COVID ward, the statistical significance was lost. Alcohol consumption showed inverse association with infection, but was above the significance threshold (*p* = 0.084), while smoking was not significant in the exploratory analysis (Table 2).

Table 3 reports the main symptoms declared by subjects who contracted the coronavirus infection (molecular test positivity). A total of 3.5% said that they were asymptomatic, while fever, fatigue, anosmia/ageusia were described by more than 50% of subjects. Over 40% of workers reported arthralgia/myalgia, cough, headache/neurological symptoms or flu-like syndrome. Less frequent symptoms included the presence of diarrhea, pharyngodynia, conjunctivitis and dyspnea or other respiratory symptoms, nausea or vomiting. Among workers positive on a molecular test (N = 116), 48.3% had been infected by a patient, while 25.0% were infected by a colleague; 15.5% stated that the infection took place within their home, while 3.5% that the contact was not a cohabitant. Among them, 10 subjects declared a double way of contagion.

## 4. Discussion

One of the aims of this study was to evaluate the percentage of contagion among workers at the Policlinico San Matteo hospital, one year after the beginning of the pandemic and two important waves of COVID-19 in Italy [14]. An increase in cumulative incidence was observed, moving from 11%, at the end of first wave, to about 17% after it [12]. The percentage of subjects who declared that they had tested positive was slightly higher than what emerged from the meta-analysis by Gómez-Ochoa et al. [15]: in that study, the prevalence of positive HCWs on a molecular test was 11%, and the average positivity to an antibody test was 7%. If we consider positivity to serological test alone, in our survey it was 12.7%, while the positivity to the molecular test was consistent with the meta-analysis (10.9%). This percentage is higher than that of the Italian population’s cumulative test positivity at the end of November 2020, i.e., just under 3% of cases were determined (about 1.5 million) [1].

Medical staff working in intensive care or first aid units were potentially most at risk of getting the infection at the beginning of the pandemic: 48.3% of workers who tested positive on a molecular test declared that they had contracted the infection from contact with a positive patient (as observed in another study [16]), while 25.0% reported having contracted the infection through work colleagues. Contacts with family members also proved to be an important vehicle for transmission [17,18]: in our survey, contagion outside the workplace, mainly attributable to a cohabitant, was 19%. These data seem to contrast with the reporting of risk perception. A possible explanation could be that infections inside the hospital date mainly to the beginning of the pandemic, while risk perception was detected at the date of the survey.

Twenty percent of subjects who got the infection reported having worked in a ward with positive COVID patients, compared to 14% of those who didn’t work in contact with infected subjects: a logistic regression model highlighted this association (OR_adj_ = 1.54). It was also observed that almost all operators declared that they had received adequate instructions for their use, which is in line with other hospital investigations [19,20]. Unfortunately, not all hospitals were able to supplying adequate equipment to their staff, especially at the beginning of the pandemic [21].

The median age of HCWs who tested positive for infection was 41 years, which is slightly lower than what was observed at the national level [22] and in our previous study [12], but is in line with American preliminary data [23] and meta-analyses [15]. In our study, age did not appear to be associated with risk of infection either in the univariate analysis or in the logistic model, and was found to be almost similar between positive and negative subjects, with no difference from other studies [24]. This lack of association was already observed in our previous study [12]. However, in the general population, age seems to be associated with both coronavirus mortality [25,26] and risk of contagion [27]. A possible explanation could be a better physical condition of HCWs compared to the general population.

Another aspect which should not be overlooked is the impact on HCWs’ mental state, due to their constant engagement during the pandemic [28,29,30]. In our survey, this aspect was not specifically studied. However, we wanted to briefly ask workers about risk perception of the infection and where they believed they felt safer. About 75% of the interviewed reported a risk perception ≥3 (median value of the data distribution), attributing an almost equal probability of contagion inside and outside the hospital. Perhaps due to their closer contact with patients, 80% of nurses/midwifes declared a risk perception ≥3: this observation doesn’t differ from what emerged in a multicenter Italian study [31] and from preliminary data relating to Chinese hospitals [32]. As mentioned above, however, we didn’t include the risk perception in the logistic model, as the answer to this question may have been influenced by having already contracted the infection and therefore it could have increased the perceived fear. The absence of difference in having contracted or not contracted the infection and the correct information about the use of PPE could be explained considering that we don’t have any information on when the infection might have occurred. As we have already pointed out, little was known at the beginning of the pandemic, therefore, information on the correct use of PPE has progressively improved.

The absence of an association between the SARS-CoV-2infection and age, sex, BMI and the possible presence of comorbidities was also observed in other studies, including a multicenter one [24]; however, these observations are not universal [33]. It must be considered that HCWs may pay significant attention to nutrition and lifestyle-based pathologies, and this could partially explain the difference from the general population [34]. In our previous study, it was instead observed how BMI and smoking seem to be associated with risk of infection [12]. A possible explanation could be the source of information, which came from the health surveillance records.

Symptomatology described by those interviewed was similar to the one of either the general or the hospitalized population [35,36], and to other investigations carried out on healthcare personnel [23,37]; symptoms such as fever, cough, loss of taste/smell and pharyngodynia were confirmed as typical of this pathology. We must consider how many of these symptoms are also characteristic of endemic infections by Adenovirus or Rhinovirus or Parainfluenza virus; therefore, even if some subjects have declared to be Clinical COVID (i.e., subjects who had symptoms compatible with the infection and contact with infected subjects) they were not considered as COVID positive subjects as they did not have a molecular or serological confirmation. We did not have information about symptom duration or severity because these questions were missing from the questionnaire.

During the pandemic, there was a debate on the potential risk associated with the use of public transportation and infection. Only about 7% of our sample reported using public transport daily, with any difference between infected and uninfected. However, it should be noticed that some workers living outside Pavia used individual means of transport; furthermore, government restrictions limited the use of public transport, and the alarming daily news may have discouraged the use of shared transportation. Having school-age children at home did not appear to be a risk factor. We need to point out that, during 2020, schools have often carried out distance learning, reducing the possibility of student aggregation and consequently the risk of becoming infected and transmitting the infection to cohabiting parents.

Finally, it has to be highlighted that, unlike what was observed in the previous analysis [12], being a current smoker would not seem to be a protective factor for infection, as evidenced by other studies in which it was assumed that chronic inflammatory disease of the respiratory epithelium hindered wall adhesion to the SARS-CoV-2 virus, thus animating a strong debate on its role [38].

*Limitations*: The study has some limitations. First of all, the questionnaire was self-reported, therefore there may have been mistakes in the interpretation of the questions. We tried to minimize this risk with staff presence in clinic sites and by using a questionnaire based on a validated model. The level of risk perception referred to the time of the questionnaire’s administration, so it may have changed during the pandemic or in relation to the infection. Some questionnaires had missing data, but not so much as to compromise the validity of the analysis. Positivity to a serological/molecular test was self-reported, so there could be a bias in detecting cases. Respondents belonged to a subgroup of workers who joined the flu vaccination campaign, and therefore they may be more sensitive to the topic of infection prevention.

## 5. Conclusions

This study confirms results observed in our hospital at the beginning of the pandemic, except for smoking and BMI. Contact with COVID-19 patients is still the principal risk factor for SARS-CoV-2 infection. The role of smoking remains controversial even in the literature; therefore, further investigations are needed.

Discrepancies observed for age and BMI, with respect to the general population, could be explained by the fact that health care workers are a selected cohort of subjects. Considering that more than 50% of workers fear a contagion inside the hospital, we believe that it is necessary to keep a high level of surveillance and that prevention strategies could be improved. The use of personal protective equipment is strongly recommended. This data collection will be useful for future surveys.

## Figures and Tables

**Table 1:** Characteristics of IRCCS Policlinico San Matteo hospital workers.

	TOTAL	COVID+	COVID−	
Sex (N)				*p* = 0.215
Male	32.5% (346)	28.6% (52)	33.3% (294)	
Female	67.5% (719)	71.4% (130)	66.7% (589)	
Age in years ^a^	42 (31–52)	42 (32–52)	42 (31–52)	*p* = 0.8119 ^b^
(N)	(1058)	(181)	(877)	
BMI (kg/m^2^) ^c^	23.6 ± 4.3	23.5 ± 4.3	23.7 ± 4.3	*p* = 0.6057 ^d^
(N)	(983)	(175)	(808)	
Smoking status (N)				*p* = 0.549
No	70.7% (749)	74.0% (134)	70.1% (615)	
Ex	8.5% (90)	7.2% (13)	8.8% (77)	
Yes	20.8% (220)	18.8% (34)	21.2% (186)	
Alcohol consumption (N)				*p* = 0.067
Yes	42.6% (448)	36.5% (66)	43.9% (382)	
No	57.4% (604)	63.5% (115)	56.1% (489)	
COVID Ward (N)				*p* = 0.009
Yes	45.4% (479)	54.2% (97)	43.6% (382)	
No	54.6% (577)	45.8% (82)	56.4% (495)	
Working Role (N)				*p* = 0.085
Physician	47.3% (504)	44.0% (80)	48.0% (424)	
Nurse/Midwife	25.2% (268)	33.0% (60)	23.6% (208)	
Social-Heath Operator	5.7% (61)	6.0% (11)	5.7% (50)	
Technician Operator	6.0% (64)	4.4% (8)	6.3% (56)	
Administrative/Researcher	15.8% (168)	12.6% (23)	16.4% (145)	
PPE use information (N)				*p* = 0.933
Yes	96.6% (1023)	96.7% (176)	96.6% (847)	
No	3.4% (36)	3.3% (6)	3.4% (30)	
Hypertension (N)				*p* = 0.576
Yes	12.5% (132)	13.7% (25)	12.2% (107)	
No	87.5% (925)	86.3% (157)	87.8% (768)	
Other Comorbidities (N)				*p* = 0.536
Yes	26.0% (277)	24.2% (44)	26.4% (233)	
No	74.0% (788)	75.8% (138)	73.6% (650)	
School-age Children (N)				*p* = 0.445
Yes	30.9% (327)	33.3% (60)	30.4% (267)	
No	69.1% (730)	66.7% (120)	69.6% (610)	
Public Transport (N)				*p* = 0.473
Yes	7.4% (78)	6.1% (11)	7.7% (67)	
No	92.6% (978)	93.9% (169)	92.4% (809)	
Physical Activity (N)				*p* = 0.132
Yes	75.9% (808)	80.2% (146)	75.0% (662)	
No	24.1% (257)	19.8% (36)	25.0% (221)	
Risk perception (N)				*p* = 0.079
0	2.1% (22)	2.9% (5)	2.0% (17)	
1	5.4% (56)	6.3% (11)	5.2% (45)	
2	17.2% (178)	17.1% (30)	17.2% (148)	
3	37.58% (389)	29.1% (51)	39.3% (338)	
4	23.2% (240)	24.6% (43)	22.9% (197)	
5	14.5% (150)	20.0% (35)	13.4% (115)	
Risk Perception (N)				*p* = 0.253
Inside the Hospital	50.2% (519)	51.2% (89)	50.1% (430)	
Outside the Hospital	47.0% (485)	44.3% (77)	47.5% (408)	
Both	2.8% (29)	4.6% (8)	2.4% (21)	

^a^: median (IQR); ^b^: *t*-test; ^c^: mean ± sd; ^d^: Mann-Whitney.

**Table 2:** Logistic Regression Model.

Logistic Regression					
	Univariate Model		Adjusted Model (N = 1041) ^a^		
	OR (95% CI)	*p*-Value	OR_adj_ (95% CI)	z *	*p*-Value
Sex				−1.03	
Male	0.80 (0.56–1.14)	0.216	0.82 (0.57–1.20)		0.305
Female	1		1		
Age (years)	0.99 (0.98–1.01)	0.746	1.00 (0.99-1.02)	0.10	0.920
Alcohol				−1.73	
Yes	0.73 (0.53–1.02)	0.068	0.74 (0.52–1.04)		0.084
No	1				
COVID Ward				2.33	
Yes	1.53 (1.11–2.12)	0.010	1.54 (1.07–2.20)		0.020
No	1		1		
Job category					
Physician	1.19 (0.72–1.96)	0.497	0.98 (0.56–1.71)	−0.77	0.941
Nurse/Midwife	1.82 (1.08–3.08)	0.026	1.37 (0.78–2.40)	1.11	0.268
Social-Heath Operator	1.39 (0.63–3.05)	0.415	1.17 (0.52–2.64)	0.38	0.704
Technician Operator	0.90 (0.38–2.13)	0.812	0.73 (0.29–1.81)	−0.69	0.493
Administrative/Researcher	1		1		

^a^ Pseudo R^2^ = 0.018; H-L χ^2^(556) = 538.77 *p*-value = 0.692; * Wald test.

**Table 3:** Symptoms of positive subjects (rt-PCR) (N = 116).

Asymptomatic	3.5% (N = 4)
Fever	62.1% (N = 72)
Cough	43.1% (N = 50)
Dyspnea/Other respiratory symptoms	9.5% (N = 11)
Pharyngitis/Pharyngodynia	21.6% (N = 25)
Conjunctivitis	13.8% (N = 16)
Diarrhea	28.5% (N = 33)
Nausea/Vomiting	8.6% (N = 10)
Arthralgia/Myalgia	48.3% (N = 56)
Asthenia	60.3% (N = 70)
Anosmia/Ageusia	53.5% (N = 62)
Headache/Neurological Symptoms	46.6% (N = 54)
Flu-like syndrome	41.4% (N = 48)

## Data Availability

The data presented in this study are available on request from the Medical Direction of the IRCCS Policlinico San Matteo, Pavia.
